# Mechanisms of Resistance and Synergy: The Role of Tumor Microenvironment in HER2-Low Breast Cancer Therapy

**DOI:** 10.3390/ph19040541

**Published:** 2026-03-27

**Authors:** Youssef Basem, Alamer Ata, Abanoub Sherif, Shaimaa Abdel-Ghany, Borros Arneth, Hussein Sabit

**Affiliations:** 1 Department of Medical Biotechnology, College of Biotechnology, Misr University for Science and Technology, Giza P.O. Box 77, Egypt; 200025117@must.edu.eg (Y.B.); 200025070@must.edu.eg (A.A.); 200033381@must.edu.eg (A.S.); 2 Department of Environmental Biotechnology, College of Biotechnology, Misr University for Science and Technology, Giza P.O. Box 77, Egypt; 3 Institute of Laboratory Medicine and Pathobiochemistry, Molecular Diagnostics, Hospital of the Universities of Giessen and Marburg (UKGM), Philipps University Marburg, Baldingerstr 1, 35043 Marburg, Germany; 4 Institute of Laboratory Medicine and Pathobiochemistry, Molecular Diagnostics, Hospital of the Universities of Giessen and Marburg (UKGM), Justus Liebig University Giessen, Feulgenstr. 12, 35440 Giessen, Germany

**Keywords:** HER2-low breast cancer, tumor microenvironment, antibody–drug conjugates, trastuzumab deruxtecan, drug resistance

## Abstract

HER2-low breast cancer, also known as IHC 1+ or IHC 2+ without ERBB2 amplification, is a new concept in the biology of breast cancer that has removed the binary classification of HER2-positive or HER2-negative breast cancer. The recent introduction of antibody-drug conjugates (ADCs), such as trastuzumab deruxtecan (T-DXd), has improved therapeutic outcomes for HER2-low breast cancer by demonstrating high efficacy in HER2-low tumors through efficient payload delivery. However, differences in ADC efficacy exist among HER2-low breast cancer patients, with tumor cells showing resistance to ADCs. Recent research indicates that the tumor microenvironment (TME) plays a critical role in determining the efficacy of ADCs against tumor cells. TME creates a barrier to the delivery of ADCs to tumor cells that show resistance to ADCs. This review article aims to highlight the current understanding of the biology of HER2-low breast cancer and its response to ADCs with reference to the tumor microenvironment.

## 1. Introduction

The historical dichotomy of breast cancer (BC) by HER2 status—strictly categorized as “HER2-positive” (IHC 3+ or IHC 2+/FISH amplified) versus “HER2-negative” has now been revised. The emergence of the HER2-low category, defined as IHC 1+ or IHC 2+/FISH-negative, has changed the classification. HER2-low tumors have distinct biological features and therapeutic opportunities [1,2]. This group accounts for about 45–55% of BC cases and forms a substantial subgroup with growing clinical significance, as recent evidence shows [3]. Whereas classic HER2-positive tumors show explicit ERBB2 amplification, HER2-low cancers display subtler transcriptomic programs and heterogeneous ERBB2 copy-number changes. These features impact drug sensitivity and guide the use of antibody–drug conjugates (ADCs) [1,4].

This reclassification shows that “HER2-negative” is not uniform, but a spectrum of biological diversity [5]. Multiple studies indicate that HER2-low tumors display higher ERBB2 mRNA and protein levels and distinct tumor–immune environments compared with HER2-0 (IHC 0). Comprehensive genomic analyses suggest that these differences often form a continuum of expression, rather than a wholly distinct molecular subtype. Nonetheless, the data support clinical non-equivalence between HER2-low and HER2-0 [1,6]. Clinically, phenotype distributions vary: HER2-low often overlaps with HR-positive luminal disease, while HER2-0 is more common in triple-negative breast cancer (TNBC) cohorts and has distinct chemosensitivity profiles [2,7,8]. Furthermore, HER2 expressions are dynamic. Paired-sample studies show that 15–36% of tumors initially labeled HER2-0 may convert to HER2-low at relapse. This highlights the value of retesting [9,10].

**Figure 1:**
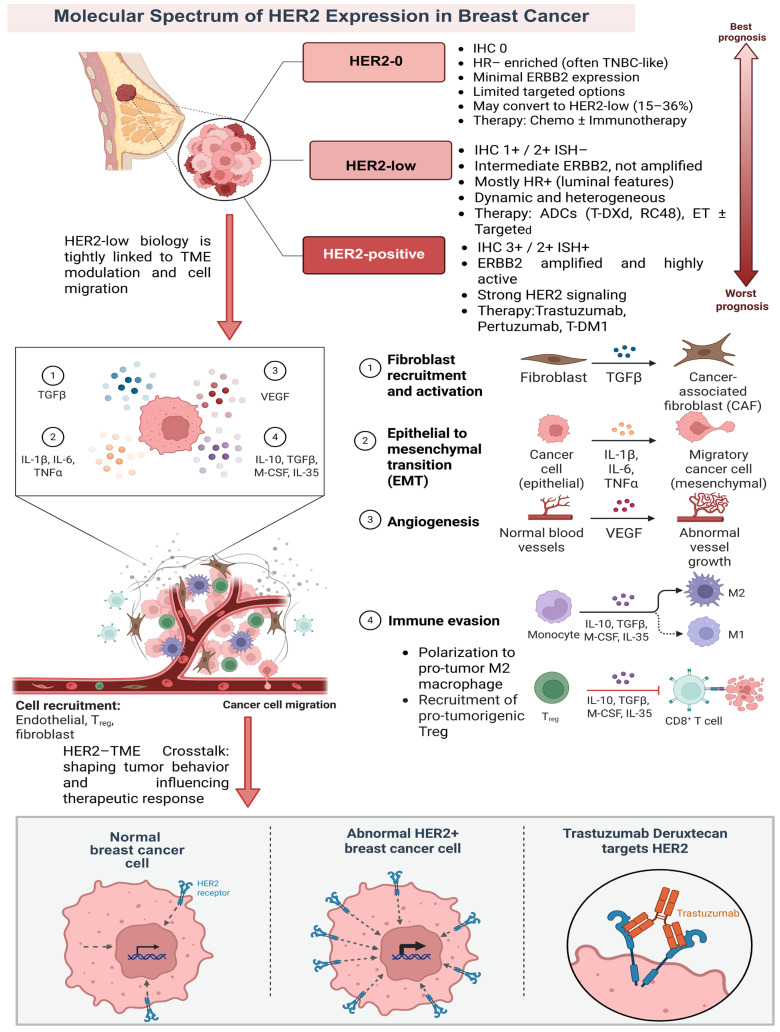
The molecular spectrum of HER2 expression in breast cancer categorizes it into HER2-0, HER2-low, and HER2-positive. These categories influence how tumors respond to treatment. The figure emphasizes the role of antibody-drug conjugates, such as trastuzumab deruxtecan, in targeting HER2-low tumors. Created in BioRender. Mahmoud, H. H. (2026) https://BioRender.com/tk00w7x, accessed on 23 March 2026).

These nuances have immediate therapeutic consequences. ADCs, such as trastuzumab deruxtecan (T-DXd), show efficacy in metastatic disease with HER2-low and even ultralow expression. This expands benefits beyond classic HER2-positive populations [11]. However, this shift creates diagnostic challenges, especially at the IHC 0 vs. 1+ threshold. Interobserver variability affects patient eligibility, underscoring the importance of better standardization and the use of digital pathology [12,13]. Multi-omics data also confirm that HER2-low tumors are distinct from HER2-0 in terms of signaling intensity, immune infiltration, and stromal architecture [14,15]. Although prognostic data about outcomes are mixed, the therapeutic implications of this spectrum continue to reshape treatment strategies [16,17,18] (Figure 1).

However, the efficacy of these precision therapies depends heavily on the tumor microenvironment (TME). The TME provides physical and metabolic sanctuaries that promote resistance [19]. Physical barriers, such as elevated interstitial pressure and a dense extracellular matrix (ECM), hinder drug penetration. Biochemical stressors, such as hypoxia and acidic pH, drive metabolic adaptations and drug efflux [20]. Mechanotransduction pathways activated by ECM stiffness, such as YAP/TAZ signaling, further improve cell survival and tolerance [21,22].

Immunosuppressive cells including tumor-associated macrophages (TAMs), myeloid-derived suppressor cells (MDSCs), and regulatory T cells create an immune-excluded phenotype. They secrete inhibitory cytokines and express checkpoint molecules such as PD-L1 [23,24]. Cancer-associated fibroblasts (CAFs) remodel the ECM and secrete exclusion factors like CXCL12, reinforcing these cellular networks. These changes create strong resistance to cytotoxic and immune therapies [19,22,25].

To overcome these barriers, therapeutic strategies must target both the TME and the tumor. Vascular normalization and mechanotherapeutics can temporarily relieve hypoxia and lower interstitial pressure. This results in brief periods during which drug delivery and T-cell infiltration are improved [26]. Reprogramming CAFs or targeting the CXCL12–CXCR4 axis can break down physical and immune exclusion. These approaches may make tumors more sensitive to checkpoint blockade [27,28,29,30].

This review aims to dissect mechanobiological layers, including vascular hypoxia, stromal mechanics, immune–myeloid gating, and metabolic constraints. The goal is to find actionable intervention windows. By linking barriers to clear biomarkers and optimizing the timing of stromal normalization with cytotoxic or immune drugs, we can turn TME resistance mechanisms into therapeutic advantages. This will help ensure that precision oncology interventions reach their full potential [11,31,32,33].

## 2. HER2-Low BC: Biological and Clinical Landscape

### 2.1. Molecular Features Distinguishing HER2-Low from HER2-Positive and TNBC

Single-cell and spatial pathology analyses show that HER2-low tumors display intermediate ERBB2 expression. They have patchy membrane staining and heterogeneous copy-number gains, which set them apart from HER2-positive and HER2-0 disease [9,34,35]. Dynamic shifts from HER2-0 to HER2-low after therapy suggest selection for subclones with residual ERBB2 signaling rather than new amplification [36]. Transcriptomic data reveal that HR+ HER2-low tumors have weaker ERBB2 pathway activity, while HER2-0 tumors are enriched for basal/TNBC signatures. This highlights different targetable biology [17,34]. Quantitative immunohistochemistry and digital scoring show that reproducibility at the 0 vs. 1+ threshold is poor, with low-end antigen density being real, but subtle and spatially variable [13,37].

Liquid biopsies can detect circulating ERBB2 clones and microenvironmental transcripts, supporting serial retesting [17,38]. Proteomics data indicate reduced levels of ERBB2 and drug-handling proteins, consistent with sensitivity to membrane-permeable ADC payloads [39]. HER2-low cells retain ligand-dependent HER family cross-talk and partial AKT/ERK phosphorylation. This supports the use of payload-driven strategies [4,15].

Brain-metastasis specimens retain low ERBB2 (also known as HER2) expression and intact antibody–drug conjugate (ADC) targetability, even with variable blood–brain barrier (BBB) physiology. This supports studies aimed at the central nervous system (CNS) [11]. Clonal modeling shows bystander-susceptible cell mosaics: micro-regions with cells expressing low levels of HER2 (1+ or 2+ by immunohistochemistry but negative by fluorescence in situ hybridization [FISH]) are mixed with ERBB2-negative neighbors. These can still be killed by diffusible topoisomerase-I payloads [15]. Overall, these data establish that HER2-low tumors are biologically distinct from HER2-0 and non-amplified compared to HER2-positive tumors. This supports treatment approaches that do not require high receptor density [34].

#### Diagnostic Implications and Assessment Challenges

Accurate assessment of HER2, a protein involved in cancer growth, is crucial for therapy selection, especially in the HER2-low and ultralow range. The College of American Pathologists (CAP) and the American Society of Clinical Oncology (ASCO) guidelines recommend standardized immunohistochemistry (IHC) scoring and in situ hybridization (ISH) confirmation for cases with uncertainty, along with interpretive reporting [40]. Discordance between IHC 0 and 1+ remains problematic. Quality con-trol, proficiency testing, and second reviews are vital for consistent results [41].

### 2.2. Epidemiology & Clinical Outcomes

HER2-low breast cancer accounts for ~50% of cases, with higher prevalence when historical IHC 0 blocks are rescored using modern assays [42,43]. It is enriched in HR+ disease and less frequent in TNBC, highlighting its epidemiologic distinction from HER2-0 [44]. Bidirectional switching between HER2-0 and HER2-low at relapse supports serial retesting for ADC eligibility [44,45]. Early-stage studies show small, context-dependent differences in PCR and survival, often modulated by HR status [46,47]. with some suggesting slightly better DFS/RFS for HR+ HER2-low tumors [48,49]. Adoption of trastuzumab deruxtecan (T-DXd) in HER2-low mBC shows meaningful real-world effectiveness [50].

In the CNS space, single-arm trials and real-world series demonstrate intracranial activity of T-DXd in HER2-low/negative cohorts, broadening its benefit to historically undertreated subgroups [51] and stereotactic radiosurgery with T-DXd maintains local control without excess radionecrosis. ADC uptake after endocrine therapy shows evolving sequences with subtle HER2-strata effects on time-on-treatment [52,53]. Neoadjuvant data indicate lower PCR for HER2-low vs. HER2-0 despite comparable long-term outcomes [54].

Health-system rescoring can reclassify IHC 0 as HER2-low, expanding eligibility and shifting survival curves through treatment access [55]. Real-world cohorts confirm T-DXd’s safety/efficacy and longer exposure compared with SG in HR+ HER2-negative, including ultralow, while TNBC outcomes are similar [52,56]. Overall, HER2-low is a high-prevalence, therapy-sensitive subgroup, emphasizing standardized scoring and dynamic retesting to optimize outcomes [44].

### 2.3. Therapeutic Landscape: ADCS, Chemotherapy, Endocrine Therapy, Immunotherapy

Early-line DESTINY-Breast06 showed significant PFS gains for HR+ HER2-low and *ultralow* (IHC > 0 < 1+) pre-chemo, expanding eligibility beyond classic low. Patient-reported outcomes from DB-06 showed maintained or improved QOL versus chemo, complementing efficacy claims in HER2-low/*ultralow* [57].

Sequence questions are emerging: a multicenter cohort of HER2-low mBC receiving both T-DXd and sacituzumab govitecan (SG) suggested activity in either order, informing post-ADC choices [58]. Datopotamab-DXd (TROP2-ADC) exhibited anti-tumor activity with manageable safety in heavily pretreated HR+/HER2− TNBC patients, providing a non-HER2 ADC option for HER2-low settings after ET [11].

For SG, the phase-3 TROPiCS-02 established OS benefit over chemo in HR+/HER2−; real-world series support the effectiveness and feasibility of T-DXd after/around T-DXd in HER2-low subsets [59]. A Nature Medicine randomized trial in Asian HR+/HER2− mBC (EVER-132-002) confirmed SG’s PFS/OS superiority vs. chemo, extending evidence to under-represented populations. Beyond T-DXd/SG, disitamab vedotin (RC48) produced responses in HER2-low mBC in phase I/Ib and multicenter real-world cohorts, with emerging signals from ESMO/AnnOnc abstracts [42].

Additional RC48 evidence includes a national real-world analysis and a retrospective monotherapy series in HER2-expressing ABC, both of which show manageable toxicity [60,61]. Combination strategies are being tested: RC48 plus anti-PD-1 (toripalimab) showed encouraging activity and acceptable safety, motivating exploration of ADC–IO combinations in low-HER2 contexts [39].

In CNS disease, a multicenter real-world study reported intracranial responses to T-DXd in HER2-positive/low patients with active brain mets; prospective phase-2 (TUXEDO-4) is underway specifically in HER2-low BCBM [51]. ASCO reports also document robust brain activity with T-DXd across HER2-positive/low, supporting systemic control in neuro-oncology settings [62].

Safety/ILD remains pivotal across ADCs; real-world pharmacovigilance cohorts emphasize protocolized monitoring to preserve benefit as use broadens [63].

In endocrine-sensitive HR+ HER2-low, ET backbones are evolving: next-gen oral SERD camizestrant improved PFS vs. fulvestrant, and AKT inhibitor capivasertib plus fulvestrant benefited HR+/HER2; both platforms can bracket ADCs in sequences [64].

Overall, today’s landscape positions T-DXd as the anchor in HER2-low/*ultralow* after ET, with SG/Dato-DXd as class alternatives, RC48 as an emerging option, and ET/targeted agents for rational sequencing while CNS-active data and standardized safety pathways extend access and durability of benefit [65]. The contemporary therapeutic landscape for HER2-low BC is summarized in Table 1.

### 2.4. Safety Considerations and ILD Risk Management with ADCS

Interstitial lung disease (ILD) and pneumonitis represent the most clinically significant adverse events associated with trastuzumab deruxtecan (T-DXd) and constitute a class-defining safety consideration 0 for HER2-low breast cancer. Across pivotal clinical trials, the overall incidence of ILD ranges from approximately 10–15%, with most events being grade 1–2; grade ≥3 events occur in a smaller proportion of patients (≈1–3%), and fatal outcomes are rare but reported. Importantly, post-approval real-world datasets suggest comparable or lower rates, reflecting heightened awareness and proactive monitoring strategies [66].

Current management emphasizes early detection through baseline and periodic chest imaging, systematic symptom assessment before each treatment cycle, and a low threshold for treatment interruption when respiratory symptoms emerge. Standardized ILD monitoring algorithms recommend immediate treatment suspension upon suspicion, prompt radiologic evaluation, and grading according to CTCAE criteria [62]. Risk mitigation relies heavily on early initiation of corticosteroids, which has been shown to reduce progression to severe or fatal ILD. For grade 1 events, temporary treatment interruption with close monitoring is generally advised, whereas grade ≥2 ILD mandates permanent discontinuation of T-DXd and systemic corticosteroid therapy [67].

**Table 1:** Key clinical trials and therapeutic strategies in HER2-low metastatic breast cancer.

Modality/Agent	Setting/Population (HER2-Low)	Core Readouts (Efficacy)	References
ADC—Trastuzumab deruxtecan (T-DXd)	Previously treated HER2-low metastatic BC (IHC 1+ or 2+/ISH−); ≥1 prior chemotherapy line; HR+ required endocrine-refractory disease	PFS (all pts): 9.9 vs. 5.1 mo; HR 0.50. OS (all pts): 23.4 vs. 16.8 mo; HR 0.64. ORR: 52.3% vs. 16.3% (TPC).	[68]
ADC—Trastuzumab deruxtecan (T-DXd)	HR-positive HER2-low mBC subgroup (DESTINY-Breast04)	PFS: 10.1 vs. 5.4 mo; HR 0.51. OS: 23.9 vs. 17.5 mo; HR 0.64.	[69]
ADC—Trastuzumab deruxtecan (T-DXd)	HER2-low “ultralow” exploratory cohort	Demonstrated clinically meaningful responses; supports HER2 expression as a continuum rather than binary.	[70]
ADC—Disitamab vedotin (RC48)	Advanced/metastatic HER2-low BC (early-phase trials & real-world cohorts)	ORR: ~30–35% (HER2-low). Median PFS: ~4–6 months (HER2-low).	[71]
Endocrine therapy ± targeted agents	HR+/HER2-low advanced BC, post-AI (HER2-low largely included within HER2-negative populations)	PFS benefit vs. ET alone: HR ~0.5–0.7 depending on agent. ORR: ~25–40% in endocrine-sensitive settings.	[72]
Chemotherapy (TPC)	ADC-ineligible patients or post-ADC progression	ORR: ~10–20% in late-line settings. Median PFS: ~3–5 months.	[73]
Immunotherapy—Pembrolizumab + chemotherapy	HR-negative/PD-L1–positive HER2-low (treated as TNBC)	PFS: 9.7 vs. 5.6 mo; HR 0.65. OS: 23.0 vs. 16.1 mo; HR 0.73. ORR: ~53% vs. 40%.	[74]
Treatment sequencing concept	HER2-low mBC across subtypes.	ADCs (T-DXd) outperform chemotherapy after standard therapies; optimal sequencing with endocrine and immunotherapy under investigation.	[75]

Multidisciplinary management involving oncology, pulmonology, and radiology, along with patient education regarding early respiratory symptoms, is central to preserving the favorable benefit–risk profile of T-DXd as its use expands in HER2-low populations.

## 3. Tumor Microenvironment in HER2-Low BC

The tumor microenvironment (TME) regulates tumor behavior and therapeutic response in HER2-low breast cancer. Interactions among immune cells, stroma, and vascular/hypoxic gradients create constraints and opportunities distinct from HER2-positive and many TNBC phenotypes [76].

### 3.1. Features of the TME Immune Cells (TILs, TAMs, MDSCs, TREGs, NK Cells)

TILs in HER2-low tumors occupy an intermediate immune niche. Bulk and spatial analyses show higher TIL density than in HER2-0 or ultralow lesions, but weaker cytotoxic programs than in many HER2-amplified tumors, so checkpoint inhibitor benefit is often variable without TME-modifying strategies [77]. TIL quality active CD8^+^ transcriptional programs and limited exhaustion correlates with durable responses and longer progression-free intervals [78].

Intra-tumor heterogeneity in CD8^+^: CD4^+^ ratios and markers of exhaustion (PD-1, TIM-3, and LAG-3) has been demonstrated by spatial and single-cell profiling, which show that these characteristics coexist within cytotoxic “islands” adjacent to immune-cold areas of the tumor, indicating that spatially-defined biomarkers for stratification may be needed [79]. SPP1-high and M2-like macrophages reside within the collagen-rich stroma of tumors, secreting matrix-remodeling enzymes and proinflammatory cytokines, creating an environment that limits T-cell entry, prevents successful ADC (antibody-drug conjugate) penetration, and establishes a macrophage-ECM (extracellular matrix) axis of resistance [80].

In addition, MDSCs functionally inhibit CD8^+^ T cells through their production of arginase and iNOS, promote angiogenesis and fibrosis, and reduce the efficacy of the ADC therapeutic payload; poor responses to checkpoint inhibitors and ADCs correlate with expansion of MDSCs as they inhibit cytotoxic effector cells and drive stromal reprogramming that hinders drug distribution [81].

Regulatory T cells (Tregs) are enriched in HER2-low microenvironments, especially in HR+ subsets; stromal TGF-β and CCL17/22 gradients favor recruitment and persistence, producing focal immunosuppression linked to shorter PFS [76].

NK cells, mediators of ADCC that can amplify antibody effects at low antigen density, are often poorly activated and excluded from desmoplastic or hypoxic niches, reducing innate ADC activity [82]. Immune populations are spatially organized: CAF-rich stromal tracks and SPP1^+^ TAM aggregates create microdomains that retain MDSCs and Tregs while excluding NK and effector CD8^+^ cells via metabolic suppression, chemokine sequestration, and ECM resistance. Multi-parametric spatial signatures combining TIL function, TAM/MDSC composition, Treg load, and NK activation are needed to identify ADC-sensitive HER2-low subsets, rather than relying on single markers [57].

### 3.2. Components of the Stroma—Fibroblasts, CAFs, and ECM

CAF heterogeneity in HER2-low tumors includes secretory pro-inflammatory fibroblasts and contractile myofibroblasts that deposit aligned collagen, increase stiffness, and impede transport and infiltration [1]. Single-cell atlases identify FAP+, myCAF, iCAF, and LRRC15+ CAF populations whose abundance governs stromal permissiveness; pro-fibrotic CAFs express LOX enzymes and TGF-β components that catalyze collagen cross-linking and create stiff, resistant niches [83].

ECM remodeling shows increased collagen I and fibronectin deposition, heightened integrin signaling, and the deposition of matricellular proteins that sequester growth factors, creating a sieve against large therapeutics and a reservoir of pro-survival signals [84]. LOX cross-linking increases ECM stiffness, activates integrin–FAK–SRC and PI3K–AKT signaling, and reduces T-cell motility, establishing a biophysical mechanism for immune exclusion and pharmacokinetic resistance [85].

There are biophysical mechanisms that create an environment that excludes immune cells and induces drug resistance [86]. The CAFs and myeloid cells would also reinforce this therapeutic resistance through reciprocal communication. In particular, CAFs are known to secrete CXCL12 and TGF-β to attract myeloid cells, and TAMs and MDSCs are known to secrete proteases and matrix remodeling factors that modify the organization of the ECM, creating a desmoplastic environment that limits the convection and diffusion of drugs and enhances the survival of cells via integrin/AKT signaling [84].

The CAFs and myeloid cells would also reinforce this therapeutic resistance through reciprocal communication. In particular, CAFs are known to secrete CXCL12 and TGF-β to attract myeloid cells, and TAMs and MDSCs are known to secrete proteases and matrix remodeling factors that modify the organization of the ECM, creating a desmoplastic environment that limits the convection and diffusion of drugs and enhances the survival of cells via integrin/AKT signaling [87].

Modulating the ECM enzymatically and inhibiting CAF activity (e.g., hyaluronidase inhibitors, LOX inhibitors, TGF-β receptor antagonists, and FAP inhibitors) has increased drug delivery in preclinical studies; however, translating these results to patients has been inconsistent and has relied on the identification of appropriate biomarkers [88].

### 3.3. Vasculature and Hypoxia

HER2-low tumors often exhibit abnormal vasculature, leading to heterogeneous perfusion and interstitial pressure, and resulting in oxygen gradients. HIF stabilization drives VEGF-driven angiogenesis, metabolic rewiring, and myeloid recruitment, which blunt immune and ADC efficacy [89].

Hypoxia upregulates PD-L1 in the tumor and myeloid compartments via HIF-1α and reprograms TAMs and MDSCs toward immunosuppressive phenotypes [51].

Vascular dysfunction impairs convective ADC transport, creating intratumoral heterogeneity in drug concentrations and enabling peripheral cells to receive sublethal exposures that select resistant clones, explaining partial ADC responses despite potent payloads [90].

Vascular normalization (e.g., using timed or low-dose anti-VEGF or anti-VEGFR agents) can transiently restore some of the vessel leakiness and interstitial pressure, improve drug and lymphocyte delivery, and reprogram the microenvironment to a permissive immune state, and potentially indicates a rationale for sequencing vascular modulators with ADCs and/or immune-therapeutics [91]. As a result, perfusion functional imaging and validated hypoxia gene signatures should be included in trial stratification to identify patients whose microvascular phenotype is expected to respond to vascular normalization before ADC delivery [92].

Hypoxia further facilitates epithelial-mesenchymal transition (EMT), increases DNA-damage repair and phenotypic plasticity, and enhances metastatic competence, biological shifts that decrease payload susceptibility and promote growth of therapy-tolerant clones in HER2-low populations [91].

### 3.4. The Microenvironment of HER2-Low Tumors Versus HER2+ and TNBC

In comparison to HER2-amplified tumors, which frequently display increased antigen density and more consistent immune activation and ADCC response, HER2-low cancers display lower HER2 expression and a less consistently inflamed TME leaving these tumors in an intermediate biological niche that creates an explanation for ADCs with high bystander activity (i.e., T-DXd) is successful in subsets, but not universally lead to durable control [33].

In contrast to many TNBCs, where high TIL numbers and neoantigen-driven immunogenicity can create ADC- and immunotherapy-sensitive microenvironments, HER2-low tumors (especially HR+ subsets) can harbor a stroma with increased CAFs, increased myeloid suppression, and hypoxia-driven metabolism to support immune exclusion rather than vigorous cytotoxic infiltration [93].

Molecular profiling indicates that HER2-low is not a single entity but a spectrum that intersects with luminal subtypes, ERBB2-adjacent signaling states, and acute proliferative programs, and that TME differences with HER2+ and TNBC are often conditional on hormone receptor status, stromal composition, and spatio-temporal hypoxia [94].

Clinical trial data (particularly DESTINY-Breast04 and mechanistic analyses from DAISY and other translational studies) show the ADC therapeutic benefit in HER2-low is regionally concentrated among tumors with favorable TME features, sufficient perfusion, less dense CAF, beneficial myeloid ratios, and functional TIL programs, applying evidence for the importance of spatial-omic biomarkers for selection of patients [53].

As a result, a systematic mechanistic combination approach offered to maximize and broaden TME responses in HER2-low disease: ADC+s with interventions to (1) decrease myeloid suppression (CSF1R/CCR2/STAT3-directed therapies), (2) transiently remodel CAF/ECM barriers (LOX/TGF-β/FAP or enzymatic strategies or approaches), and (3) normalize vascular barriers for enhanced perfusion and immune access, with repeated spatial biopsies included as trial endpoints [95].

In conclusion, HER2-low tumors shape a complex, spatially organized microenvironment characterized by intermediate TILs, pro-fibrotic CAFs, SPP1-enriched TAM niches, MDSC-mediated immune suppression, and perfusion-limited hypoxic domains; only by measuring and targeting these interdependent axes will we be able to leverage mechanistic insights into reproducible clinical benefit to the large HER2-low population [96] (Table 2).

This table outlines various combination strategies involving antibody-drug conjugates (ADCs) and tumor microenvironment (TME) targeting agents. It includes the mechanisms of synergy, the role of the TME, and the key benefits or findings observed in enhancing ADC effectiveness across different therapeutic combinations, including T-DXd and immune-modulatory agents.

## 4. TME-Mediated Mechanisms of Resistance

The tumor microenvironment (TME) is not merely a passive, homogeneous environment for tumor cells. Rather, the TME is an active, dynamic ecosystem whereby tumor progression and therapeutic resistance are promoted and sustained. In HER2-low breast cancers, a biologically heterogeneous subtype recently embraced as a therapeutic application with agents such as trastuzumab-deruxtecan, resistance emerges not solely through the altered biology of the target antigen but through TME-mediated.

**Figure 2:**
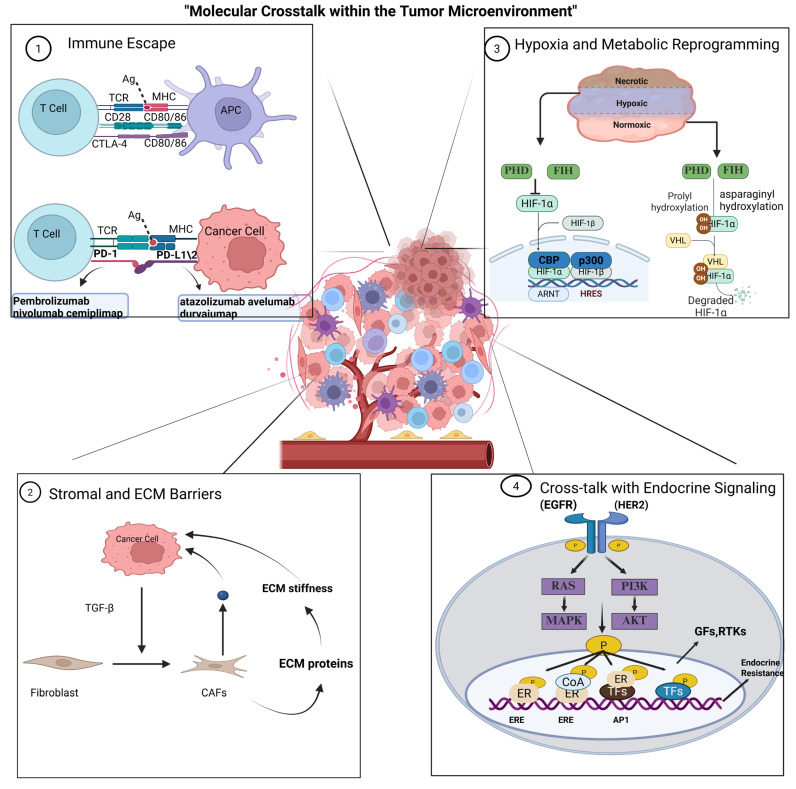
Integrated overview of the major molecular interactions shaping the tumor microenvironment, highlighting immune escape mechanisms, stromal and ECM remodeling, hypoxia-driven metabolic reprogramming, and cross-talk with endocrine signaling pathways that collectively sustain tumor progression and therapeutic resistance. Created in BioRender. Mahmoud, H. H. (2026) https://BioRender.com/7x3q0dz, accessed on 23 March 2026.

Mechanistically, therapeutic efficacy is constrained by impaired drug delivery, blunted immune detection, metabolic rewiring, and the activation of compensatory signaling networks. These resistance mechanisms co-exist and overlap spatially and temporally, giving rise to patterns of primary insensitivity and acquired relapse that are distinct from those observed in classical HER2-positive disease [105] (Figure 2).

### 4.1. Immune Escape Immune Escape Within the TME

Describes a coordinated series of methods that collectively keep tumor cells less visible or less susceptible to the immune effectors that attack mutant cells. Immune escape is especially pertinent to HER2-low tumors, given that ADC therapy (which induces immunogenic cell death and bystander killing) and immune checkpoint inhibitors (ICIs) rely heavily on pre-existing or inducible anti-tumor immunity. When the TME is “cold” (low neoantigen burden and sparse CD8^+^ infiltration), both ADC and ICI benefits are diminished [106].

Mechanistically, the upregulation of inhibitory immune checkpoints (PD-L1 in tumor and myeloid elements, PD-1, CTLA-4 on T cells) serves as an adaptive response to inflammatory stress or metabolic stress and suppresses effector T cell function. Hypoxia and hypoxia-inducible factors (HIF) signaling enhance PD-L1 transcription within tumor and stromal cells and link metabolic stress to standard immune checkpoint targets. Clinically, elevated PD-L1 and decreased CD8^+^ TIL density correlate with reduced responses to ICIs and decreased progression-free survival in BC patients across several cohorts [107]. In addition to checkpoint molecules, the TME supports the recruitment and expansion of a regulatory population, namely MDSCs, Tregs, and M2-polarized macrophages, which secrete soluble suppressive mediators (TGF-β, IL-10, arginase, iNOS) to diminish local cytotoxicity and antigen presentation.

For instance, MDSC/Treg accumulation is associated with significant decreases in CD8^+^ proliferative indices and decreased IFN-γ production in ex vivo assays; these immunosuppressive pockets are often co-localized with hypoxic, ECM-rich areas [108]. A particularly detrimental pattern of ADC efficacy occurs when there is immune exclusion and poor ADC penetration (see Section 4.2): ADCs may kill perivascular tumor cells and release neoantigens; however, if effector cells cannot access the deeper tumors or are suppressed, then any immunogenic cell death does not produce an effective anti-tumor immune response, leading to a temporary decrease in tumor volume followed by re-growth. Correlative analyses from recent trials with T-DXd indicate that benefit is enriched in patients with higher baseline TILs, highlighting that long-term responses depend on an intact anti-tumor immune-axis [109].

Therapeutic strategies should therefore combine ADCs with measures to relieve local immune suppression, restore effector function, and reverse stromal or metabolic constraints. Selection of biomarkers (PD-L1, spatial TIL mapping, myeloid signatures) must guide combination selection, as ICI + ADC benefit is confined to biomarker-defined subsets [110].

### 4.2. Barriers of Stroma and ECM

The physical and biochemical properties of the stroma are among the major determinants of macromolecular delivery. Cancer-associated fibroblasts (CAFs) deposit and arrange fibrillar components (collagen I/III, fibronectin) and glycosaminoglycans (especially hyaluronan), which increase interstitial fluid pressure (IFP), stiffen the ECM, and act as barriers to convective/diffusive distributions of antibody and ADCs. Single-cell and spatial transcriptomic data have shown that CAFs exhibit subtypes (inflammatory, myofibroblastic, and antigen-presenting CAFs) with distinct secretomes that may influence drug penetration and immune access differently [111].

Biophysical studies demonstrate that desmoplastic niches can reduce ADC penetration to sub-millimeter distances, often substantially decreasing macromolecular delivery compared with well-perfused regions [112].

CAFs and myeloid cells also secrete MMPs and matricellular proteins that sequester payloads, alter linker cleavage kinetics, or reroute ADC endocytosis, while CAF-derived TGF-β enforces local immunosuppression; together, these mechanisms create dual pharmacokinetic and pharmacodynamic resistance to ADCs [111]. Preclinical stromal modulation (hyaluronidase, LOX inhibition, TGF-β blockade, or FAP targeting) has increased intratumoral ADC concentrations and improved responses in select models, but randomized clinical benefits have been inconsistent and demand biomarker-based selection [86].

Actionable recommendations (stromal barrier): studies should include tumor PK endpoints while on treatment (e.g., microdialysis or spatial mass spec), histo-spatial quantification of ECM, and CAF phenotyping. From a drug-design perspective, next-generation ADCs with enhanced payload potency, cleavable linkers designed for streaming enzymes, or smaller binding scaffolds, could also partially solve binding-site issues [113].

### 4.3. Hypoxia and Changes in Metabolism

Hypoxia is present in all solid tumors and capitalizes on widespread, HIF-dependent and HIF-independent, tumor and stromal reprogramming. Upon activation of HIF-1α/HIF-2α, genes for angiogenesis (VEGF), glycolytic enzymes (e.g., LDHA), and survival factors are induced, and concurrently, expression of drug-efflux transporters and DNA repair programs increases, thereby enhancing resistance to cytotoxic agents. Importantly, hypoxia promotes immune suppression by consequent increases in PD-L1 expression, myeloid cell suppression induced by recruitment, and alterations in antigen presentation [114].

Metabolically, many HER2-low tumors exhibit increased glycolysis and elevated extracellular lactate levels. Clinically, tumor lactate concentration measures indicate values several-fold greater than surrounding normal tissue (often ~5–20 mM in tumors versus ~1–2 mM in adjacent normal interstitium), and research demonstrated that such levels inhibit proliferation and secreting cytokines of CD8^+^ T-cells, promote polarization of macrophages into M2 phenotypes, and impair dendritic cell activation, all of which detriment innate and adaptive, anti-tumor immunity. Thus, lactate is not simply a metabolic waste product in solid tumors, but alternatively functions as a paracrine immunoregulatory signal through MCT transporters and GPR81 [115].

Changes in metabolism induced by hypoxia also affect ADC biology: representations of acidic, hypoxic microenvironments can alter linker cleavage, payload stability, and intracellular trafficking; in addition, hypoxia-related suppression of apoptosis and enhancement of survival pathways may make residual cells less sensitive to cytotoxicity associated with payloads. Preclinical studies utilizing combination strategies—e.g., LDH inhibitors, MCT inhibitors, antagonists of HIF, or buffering agents to modulate pH—have been able to restore T-cell function and enhance the efficacy of ADCs in mouse studies, in some cases achieving improvements of tumor growth delay of 2-fold to 4-fold compared to ADC alone. While these improvements are promising, caution must be exercised when defining toxicity, as normal tissues are also affected by metabolic inhibitors [116].

In a translational context, measures of hypoxia (pimonidazole staining and HIF target gene signatures), proxies of lactate (MRS imaging and lactate in blood or from biopsies), and imaging of different pH levels may be utilized to stratify patients and guide interventions targeting metabolism. As a proof-of-concept, combinations of ADCs with metabolic targets or immunometabolism interventions should be evaluated first in patients with apparent or demonstrated hypoxic/metabolic representation [117].

### 4.4. Cross-Talk with Endocrine Signaling (ER/HER2)

Reciprocal interaction between ER and HER family pathways comprises a principal adaptive mechanism in HR+/HER2-low disease. Even low or heterogeneous HER2 expression can nonetheless activate downstream PI3K/AKT and RAS/MAPK cascades that bypass ER inhibition, while endocrine therapy can provoke compensatory RTK upregulation and downstream pathway activation, producing reciprocal plasticity and sequencing complexity [118].

In addition to estrogen signaling via estrogen receptors, steroid hormones may interact with HER family pathways and influence tumor behavior. For example, the androgen receptor (AR) has been shown to interact with HER2 signaling pathways and may modulate downstream effects, including pathways associated with cell proliferation and survival [119]. Glucocorticoid receptors (GR) also play a role in context-dependent adaptive responses that improve cellular tolerance to stress and, in certain situations, potentially contribute to the development of therapeutic resistance. The mineralocorticoid receptor (MR) has not been as widely studied in breast cancer; however, it has also been identified in transcriptional patterns associated with metabolic regulation and intracellular signaling. Together, these steroid hormone receptors present a broader endocrine regulatory environment, considering how they may impact the dynamics of HER2-mediated signaling and treatment response [120].

Activating PIK3CA mutations and PTEN loss amplify PI3K output and are associated with reduced responsiveness to endocrine agents and some anti-HER2 approaches, supporting biomarker-guided multi-node targeting in selected patients [111].

Clinically, combinations of endocrine agents with CDK4/6 inhibitors, PI3K inhibitors, or HER2-directed ADCs yield benefit in selected contexts, but cross-trial comparisons reveal variable magnitude of effect and added toxicity; genomic and pathway-activation signatures should, therefore, guide combination selection to maximize net clinical benefit [121].

### 4.5. Integrated Viewpoint and Prioritization for Translational Strategy

The interdependence of these resistance mechanisms is apparent in that hypoxic microenvironments occur mainly within stroma-laden tissue and are immune-excluded, while metabolic adaptations are often found within the context of endocrine bypass pathways. Therefore, increasing the dose of a single modality alone will not usually result in durable eradication in most HER2-low tumors. In particular, a translational framework that is based on a comprehensive baseline assessment of the tumor microenvironment (i.e., PD-L1 status and TIL spatial mapping, hypoxia status, and lactate levels as well as scoring of ECM/CAF, and routine genomic abnormalities such as PIK3CA, TP53, and PTEN), tumor pharmacokinetics endpoints from an ongoing assessment through multiple surgical biopsies, and adaptive trial designs for biomarker-based cohort expansion [106].

Prioritized concepts include myeloid suppression relief (CSF1R, CCR2, STAT3 inhibitors), transient CAF/ECM remodeling (LOX, TGF-β, FAP or enzymatic methods), and vascular normalization to improve perfusion and immune access; these must be coupled to spatial-omic endpoints to document ecosystem perturbation and select responsive subgroups [122].

Metabolic/immunometabolic co-targeting—the combination of ADCs with agents that decrease tumor lactate or antagonize HIF programming associated with hypoxia in tumors with a biomarker support and work together to reverse T-competence in T-cells and sensitizing the T-cells to payload, although early models have shown 2–4× efficacious measures, but clinical safety sequencing and mapping must be cautiously considered [123].

Ultimately, rational endocrine combinations—in HR+ and HER2-low disease—pairing ADCs or HER2 pathway inhibitors with ER pathway blockers, selectively with PI3K and ultimately CDK4/6 inhibitors, all guided by genomic evidence of pathway activation to stave off rapidly adaptive bypasses, and such are able to (latch onto repertoire creating broad population control during treatment) [118].

Finally, early-phase trials should mandate spatially resolved correlative science (multiplex IHC, spatial transcriptomics, tumor microdialysis, or mass spec for PK) and adaptive designs that allow biomarker-driven cohort expansion. Only by treating the tumor as an ecosystem and by carefully quantifying how interventions perturb that ecosystem can we convert the profound activity of agents such as T-DXd into durable patient benefit in the heterogeneous landscape of HER2-low BC [2].

## 5. Synergistic Interactions: Turning TME into a Therapeutic Ally

### 5.1. Antibody-Drug Conjugates (ADCS) and Bystander Effect

#### 5.1.1. Mechanism of Trastuzumab Deruxtecan

**Figure 3:**
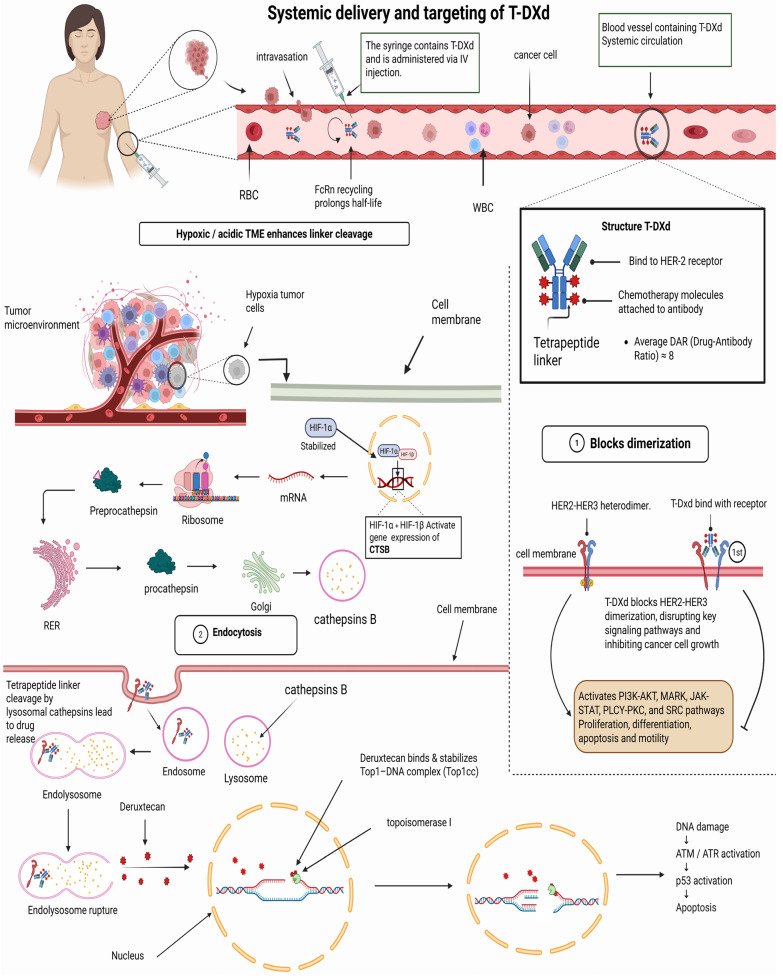
T-DXd is given IV, binds to HER2 on cancer cells, is internalized, and its linker is cleaved in the lysosome, releasing deruxtecan. The payload inhibits Topoisomerase I, causing DNA double-strand breaks and apoptosis, while also blocking HER2–HER3 signaling. Created in BioRender. Mahmoud, H. H. (2026) https://BioRender.com/elyuxvj, accessed on 23 March 2026.

Trastuzumab deruxtecan (T-DXd) is an antibody-drug conjugate (ADC) designed to kill cancer cells that make too much HER2 (Figure 3). It consists of an anti-HER2 antibody linked, via a cleavable linker, to a potent topoisomerase I inhibitor (deruxtecan). Once the antibody binds to HER2 on the tumor cell surface, the complex is taken up into the cell. Inside, the linker is broken down by lysosomal enzymes or, in some cases, by proteases outside the cell, releasing the membrane-permeable cytotoxic drug. This payload then spreads within the tumor cell, causing DNA damage and ultimately cell death [97].

An essential attribute of T-DXd is its bystander effect, allowing the drug to exit the target cell and kill adjacent tumor cells with low or absent HER2 expression [78]. In contrast, earlier ADCs such as trastuzumab emtansine (T-DM1) employ a non-cleavable linker and, therefore, act only on cells with high HER2 expression [15]. The strong bystander effect of T-DXd enables it to counter tumor heterogeneity by targeting HER2-low or HER2-negative cells adjacent to HER2-positive cells, which partly accounts for its effectiveness in HER2-low breast cancers [33].

#### 5.1.2. Role of TME in Facilitating the Bystander Effect

The tumor microenvironment (TME) plays a key role in the distribution of ADC payloads. Proteases in the TME, such as the cysteine protease cathepsin L, can enhance the bystander effect of T-DXd by cleaving its linker extracellularly, releasing the drug even without full internalization. This allows T-DXd to target adjacent HER2-low or HER2-negative cells, circumventing the need for direct antigen binding [124].

Adding active cathepsin L to experimental models made T-DXd much more effective at killing HER2-negative tumor cells, whereas T-DM1 had no effect. This shows that an active tumor microenvironment can enhance ADC efficacy [124]. On the other hand, certain features of the tumor microenvironment can restrict the extent of the bystander effect [125].

A strongly fibrotic stroma or high interstitial pressure may physically block drug transport, whereas acidic or oxygen-poor areas can reduce drug effectiveness [126]. Excess cancer-associated fibroblasts (CAFs) that produce collagen and hyaluronan create a structural barrier that diminishes ADC penetration, leading to reduced uptake of large therapeutic molecules and potential treatment resistance [127].

The tumor microenvironment (TME) is a double-edged sword: proteases and permeable blood vessels can facilitate wider diffusion of cytotoxic payloads, whereas a dense stromal network or extracellular binding can trap the drug and limit its activity [6]. Understanding these processes has led to strategies such as combining treatments with catalysts or agents that modify stromal structure to enhance secondary effects in solid malignancies [128].

### 5.2. Immunotherapy Plus HER2-Targeted Therapy

Combining PD-1/PD-L1 blockers with HER2-targeted ADCs is a promising strategy to enhance anti-tumor immunity. Cell death induced by these ADCs can be highly immunogenic, releasing tumor markers and stress signals that activate immune cells in the TME [97]. T-DXd, through its payload Deruxtecan (DXd), elicits stronger immunogenic cell death (ICD) than T-DM1, as evidenced by increased release of DAMPs that activate nearby myeloid cells via TLR4 and STING signaling pathways [127].

The antibody component of T-DXd engages Fcγ-receptors to enhance antibody-dependent cellular phagocytosis (ADCP), like trastuzumab. The combination of DXd-induced ICD and ADCP promotes greater tumor antigen uptake by macrophages and stronger activation of antigen-specific CD8^+^ T cells than T-DM1 [97]. T-DXd can convert immunologically “cold” tumors into more inflammatory and reactive microenvironments. However, this approach may also trigger immune-suppressive mechanisms; for example, in gastric cancer, T-DXd has been shown to increase PD-L1 expression in surviving tumor cells via interferon and DNA-damage response pathways [76]. Likewise, T-DXd-induced tumor destruction triggers the expression of the “do n’t-eat-me” signal CD47 on cancer cells, functioning as a reactive immune evasion mechanism [97].

These results strongly support combining ADCs with checkpoint inhibitors, as ADCs enhance immune recognition of tumors while checkpoint blockade prevents immune evasion. In HER2-driven preclinical models, combining T-DXd with anti-PD-1 or anti-PD-L1 antibodies significantly increased T-cell infiltration and improved cure rates compared with T-DXd alone [129].

Combining T-DXd with anti-PD-1 treatment resulted in a more significant decrease in tumor size and a greater activation of CD8^+^ T-cells. This shows a synergistic effect, where ADC-triggered immunogenic cell death enhances the efficacy of checkpoint inhibition on reactivating T-cells. Current clinical investigations are assessing this strategy, including the DESTINY-Gastric03 trial, which is testing T-DXd alongside pembrolizumab or durvalumab in patients with HER2-positive and HER2-low cancers [97]. Preliminary clinical findings suggest potential advantages of combining immunotherapy with HER2-targeted treatments, although results appear strongly influenced by patient selection. In the phase II KATE2 trial, patients with advanced HER2-positive BC were treated with T-DM1 (ado-trastuzumab emtansine), either alone or in combination with the PD-L1 blocker atezolizumab. However, the trial did not achieve its primary progression-free survival goal in the unselected patient population [130].

In PD-L1–positive tumors, adding a checkpoint inhibitor nearly doubled median progression-free survival (8.5 vs. 4.1 months) and improved response rates (54% vs. 33%) compared with T-DM1 alone. Patients with high baseline tumor-infiltrating lymphocytes (TILs) also showed better outcomes (median PFS 8.5 vs. 5.3 months) [131].

No improvements were observed in PD-L1–negative tumors, highlighting the importance of the immune landscape, particularly PD-L1 expression and TILs, in evaluating immunotherapy efficacy in HER2-positive malignancies [98]. This underscores why an immunogenic ADC like T-DXd, which induces inflammation in the tumor microenvironment, is considered a promising partner for checkpoint inhibitors, and ongoing trials are exploring this combination in both HER2-positive and HER2-low patients.

In addition to PD-1/PD-L1, other checkpoint molecules and innate immune modulators are being investigated [97]. For example, preclinical studies have demonstrated that inhibiting the macrophage checkpoint CD47, which is upregulated after T-DXd treatment, boosts the phagocytosis of tumor cells and promotes long-lasting anti-tumor CD8^+^ T-cell responses when combined with T-DXd [99].

To conclude, combining HER2-targeted ADCs with immunotherapies provides a potent dual mechanism: the ADC facilitates the release of tumor antigens and immune activation within the tumor microenvironment, while the checkpoint inhibitor activates T-cell responses. Together, this approach converts an immunosuppressive tumor environment into a catalyst for efficient tumor elimination [39].

### 5.3. Endocrine Therapy Combined with HER2-Low Targeting

Since most HER2-low breast cancers are hormone receptor–positive, there is strong rationale for combining HER2-targeted ADCs with endocrine therapy in this subgroup [15]. Traditionally, HR-positive/HER2-negative breast cancers have been treated with endocrine therapy, yet resistance almost inevitably develops, frequently via growth factor pathway activation and modulation of the tumor microenvironment. In HER2-low disease, subthreshold HER2 activity or alternative receptor tyrosine kinases may serve as escape mechanisms once estrogen receptor signaling is inhibited [97]. Combining ER-targeted therapy with a HER2-directed ADC offers a method to surmount this adaptive resistance by eliminating hormone-independent clones and newly formed HER2-driven subpopulations.

In HR-positive metastatic breast cancer resistant to hormonal therapy, trastuzumab deruxtecan significantly improved progression-free survival compared with physician-chosen chemotherapy [15]. ADCs can counter endocrine resistance driven by the tumor microenvironment, where cancer-associated fibroblasts (CAFs) impair estrogen receptor (ER) signaling via paracrine effects, reducing ER-α expression and promoting estrogen-independent, therapy-resistant phenotypes [132].

These tumor microenvironment–driven changes explain why some HER2-low, ER-positive tumors become resistant to hormonal therapy despite continued ER expression. The use of HER2-targeted ADCs provides a strategy to eliminate tumor cells supported by the stroma or those that have shifted toward ER-independent survival pathways [133]. By targeting ER-independent subclones, ADCs reduce overall tumor burden and enrich hormone-sensitive cells that are more likely to respond to endocrine therapy. Early clinical studies suggest that sequential ADC administration after the development of endocrine resistance can improve patient outcomes, although the optimal timing of ADC–endocrine combinations remains under investigation [15].

### 5.4. Anti-Angiogenic and Stromal Targeting Approaches

#### 5.4.1. VEGF Inhibition and Vascular Normalization

In HER2-low breast cancer, tumor vasculature is often abnormal, limiting drug delivery and oxygenation. Targeting vascular endothelial growth factor (VEGF) aims to normalize tumor blood vessels and improve therapeutic efficacy. Studies investigating anti-VEGF monoclonal antibodies have shown improved vascular architecture, reduced vessel leakage, and enhanced delivery of chemotherapeutic agents and ADCs to tumor cells. Preclinical and early-phase clinical data suggest that VEGF inhibition may facilitate ADC penetration by improving vascular permeability; however, robust clinical evidence supporting this strategy remains limited [134].

Also, stopping VEGF has been proven to make tumors less hypoxic, which is a big problem for chemotherapy and immunotherapy.

VEGF inhibition stabilizes blood arteries, which reduces hypoxia and increases the oxygen flow to tumor cells. This makes other treatments work better. It has also been noted that using VEGF inhibitors in HER2-low tumors enhances oxygenation and elicits a more robust tumor response to treatment [135]. VEGF inhibitors help malignancies get around resistance mechanisms. Research has demonstrated that combining VEGF inhibitors with chemotherapy not only stabilizes the tumor vasculature but also lowers tumor interstitial pressure, thereby improving delivery and boosting the overall efficacy of treatment [136].

#### 5.4.2. Targeting CAFs to Improve ADC Delivery

CAFs are vital elements of the tumor microenvironment and contribute significantly to the development of drug resistance across multiple cancer types, including HER2-low breast cancer. CAFs facilitate the formation of a dense extracellular matrix, which serves as an obstacle to drug delivery, preventing ADCs from effectively reaching the tumor [127]. It has been stressed that targeting CAFs using hyaluronidase, an enzyme that breaks down ECM components, makes it easier for trastuzumab deruxtecan to reach HER2-low BC cells, which makes the treatment more successful [137]. Moreover, fibroblast activation protein (FAP), which is highly expressed on CAFs, has been identified as a prospective therapeutic target. A recent study demonstrated that targeting FAP+ CAFs with a monoclonal antibody markedly improved the delivery of trastuzumab emtansine to HER2-low tumors, indicating that targeting CAFs can boost the therapeutic effectiveness of ADCs [42].

Beyond extracellular matrix (ECM) remodeling, cancer-associated fibroblasts (CAFs) secrete growth factors that promote tumor progression and therapeutic resistance. Targeting CAFs reduces these signals and may enhance responsiveness to HER2-targeted therapies. In HER2-low models, combining CAF-targeting agents with VEGF inhibitors improved drug delivery and increased the efficacy of trastuzumab deruxtecan [138]. Enzymatic ECM modulation using collagenase disrupts collagen fibers, facilitating ADC penetration; co-administration of collagenase and VEGF inhibitors further enhanced ADC delivery and treatment outcomes in HER2-low breast cancer [139]. Collectively, dual targeting of VEGF signaling and CAFs represents a promising strategy to overcome resistance, with ongoing clinical trials evaluating these combinations, including T-DXd with TME-modulating approaches [140].

## 6. Emerging Biomarkers from the TME in HER2-Low BC

HER2-low breast cancer, defined by immunohistochemistry (IHC) scores of 1+ or 2+ without ERBB2 amplification, constitutes a significant proportion (about 40–60%) of cancers formerly categorized as HER2-negative. This subgroup has become increasingly important because novel HER2-directed antibody drug conjugates, such as trastuzumab deruxtecan (T-DXd), have shown significant clinical benefit in patients with HER2-low metastatic disease [57].

Like other breast cancer subtypes, HER2-low tumors develop within a complex tumor microenvironment (TME) composed of immune cells, stromal elements, and extracellular components that critically influence tumor biology and therapeutic response. Extensive evidence links the TME to breast cancer progression and therapy resistance [141]. Tumor-infiltrating lymphocytes (TILs), together with immune-related gene expression signatures, are considered valuable predictive and prognostic biomarkers, especially in more aggressive forms of BC such as HER2-positive and triple-negative subtypes [53]. In contrast, the immune landscape of HER2-low tumors has only recently begun to be characterized. Emerging data suggest distinct TME features with potential biomarker value. The following sections summarize recent studies on TME biomarkers in HER2-low breast cancer, focusing on TILs, immune gene signatures, spatial and single-cell profiling, and liquid biopsy approaches including circulating tumor DNA and exosome analysis [142]. Table 3 summarizes emerging biomarkers like TIL density, immune gene signatures (e.g., PD-L1, TIGIT), spatial features (e.g., TLS density, CAF subtypes), and liquid biopsy markers (e.g., ctDNA mutations, exosomal PD-L1/miRNAs).

### 6.1. TILs and Immune Gene Signatures in Her2-Low Cancers

The density of TILs and immune gene expression profiles are pivotal biomarkers of the TME in BC. In TNBC, for instance, higher TIL levels are linked to substantially better survival rates [139]. In HER2-positive breast cancer, TIL levels serve as a predictive marker for the benefit from both chemotherapy and targeted therapies [150]. On the other hand, tumors with low HER2 expression are more commonly hormone receptor-positive and show reduced immunogenicity. Studies have shown that, generally, immune activity is lower in HER2-low cancers compared to HER2-zero (IHC 0) tumors. Furthermore, it has been observed that HER2-low tumors exhibit significantly lower tumor-infiltrating lymphocyte (TIL) density, indicating a weaker immune response relative to HER2-zero tumors [6].

In clinical practice, TIL levels in HER2-low breast cancer resemble those of ER-positive luminal tumors rather than highly immune-inflamed subtypes. Ko et al. analyzed 395 immune-related genes and observed no overall difference in immune gene expression between HER2-low and HER2-zero tumors. However, stratification by ER status revealed that HER2-low/ER-positive tumors exhibit markedly lower PD-L1 and TIGIT mRNA expression compared with HER2-zero/ER-negative cancers [144].

This is in line with the immunological microenvironment that is usually “colder” in luminal breast tumors. Similarly, Sun et al., in a cohort of early-stage HER2-low patients, found that high stromal TIL levels (>10%) were uncommon; however, when present, they were strongly associated with improved outcomes. In multivariate analysis, patients with TIL-high HER2-low tumors had an approximately 0.47 hazard ratio for disease-free survival compared to those with TIL-low tumors [143]. From what the studies show, HER2-low tumors don’t really attract many TILs, basically looking a lot like HER2-0 in that sense, and they also have weaker expression of immune checkpoints. Still, in the rare cases where TIL levels are high, patients in this group seem to do noticeably better in terms of prognosis [53].

Looking past TILs, researchers have also looked at wider immune gene signatures in HER2-low cancers. The takeaway from gene expression studies is that it’s really the hormone receptor status that drives immune activity, not the HER2 level itself. Shen et al. found that interferon and T-cell–related gene signatures were more pronounced in all HR-negative subtypes, whether HER2-low or HER2-high, while HR-positive groups showed much weaker immune activity [128].

Overall, HER2-low/ER-positive tumors behave similarly to luminal breast cancers, with only moderate immune gene expression, whereas HER2-low/ER-negative tumors cluster with more immune-active subtypes. Several studies have identified tumor microenvironment (TME)–related gene modules associated with clinical outcomes. Notably, a prognostic TME signature derived from The Cancer Genome Atlas demonstrated that high-risk patients, characterized by elevated expression of specific TME genes, exhibited reduced cytotoxic lymphocyte infiltration and poorer survival [105]. On the flip side, TME signatures linked to better outcomes were those associated with stronger infiltration of CD8^+^ T cells, NK cells, and other antitumor immune cells [100]. All this highlights how combined immune gene signatures can help sort patients into different risk groups, and using the same strategy could point us toward TME biomarkers that are specific to HER2-low disease.

In addition to TILs, other immune cell populations have been proposed as biomarkers.

Tumor-associated neutrophils (TANs) are becoming more recognized as unfavorable prognostic factors in breast cancer. Elevated levels of TANs are often correlated with worse outcomes, as these cells, especially the pro-tumor N2 subtype, stimulate angiogenesis and facilitate tumor invasion. Clinical findings indicate that heightened TAN density is associated with diminished overall survival and an elevated probability of relapse [151].

In HER2-low tumors, which are usually ER-positive and have fewer immune cells, increased levels of TANs or neutrophil-related signals in the TME may also mean worse outcomes. Angiogenesis represents another prognostic factor, as BC studies have shown that increased microvascular density, a marker of active angiogenesis, is linked to reduced survival [33]. Because some HER2-low tumors develop in older, hormone-rich settings, they may be especially prone to stronger angiogenic activity or greater TAN infiltration, making these factors potential TME biomarkers. In summary, immune microenvironment markers like TIL density, interferon and T-cell gene signatures, neutrophil profiles, and angiogenesis markers are becoming important tools for predicting and diagnosing HER2-low BC [99].

### 6.2. Spatial Transcriptomics and Single-Cell Profiling of the TME

New single-cell and spatial profiling technologies enable detailed mapping of the breast cancer microenvironment, revealing the organization of immune, stromal, and tumor cells and uncovering spatial biomarkers. Combining single-cell RNA sequencing with spatial transcriptomics has identified unique cellular niches, including rare populations at the tumor periphery, and highlighted myoepithelial boundary cells that restrict tumor growth [6]. In HER2-positive and TNBC models, spatial analyses using Visium transcriptomics revealed localized tertiary lymphoid structures (TLS) enriched in T cells, macrophages, and type I interferon activity, associated with higher immune gene expression and potential antitumor activity [145].

Single-cell RNA sequencing (scRNA-seq) is an important approach for finding biomarkers and mapping TME diversity. In ER-positive breast cancers, comparisons between primary tumors and their matched metastases showed that metastatic sites exhibited an immunosuppressive TME profile. These lesions were notably enriched with CCL2^+^ macrophages, exhausted PD-1^+^ CD8^+^ T cells, and FOXP3^+^ regulatory T cells, along with a widespread decrease in tumor–immune cell interactions [152]. These changed cell states, like M2-like macrophages or tired T cells, can be molecular signs of a hostile TME. In a separate single-cell investigation focusing on the invasive “interface zone,” the boundary between tumor tissue and adjacent normal tissue, researchers identified this area as densely populated with very pro-tumor cell types. These encompassed regulatory T cells, M2 macrophages, pro-angiogenic mast cells, fibroblasts synthesizing collagen, and highly proliferative epithelial cells [125]. This so-called “tumorigenic niche” is defined by ligand-receptor signaling pathways, like IL-6/STAT3, that help the tumor avoid the immune system. In support of this, exosomes from BC that carry the IL-6 receptor β-chain (gp130) have been demonstrated to activate STAT3 in macrophages, pushing them toward a condition that helps them survive and suppresses the immune system [148]. All these pieces of information point to several possible TME biomarkers. For example, there are cell-type-specific signature scores for regulatory T cells, M2 macrophages, or cancer-associated fibroblasts (CAFs). There are also geographical features like the density of tertiary lymphoid structures (TLS) or the prevalence of direct myeloid T cell contacts.

### 6.3. Liquid Biopsy: CtDNA and Exosomes Reflecting TME Activity

Liquid biopsy techniques, such as circulating tumor DNA (ctDNA) and extracellular vesicles (exosomes), offer a non-invasive method charged into the bloodstream, encompassing tumor-specific modifications such as mutations, copy number variations, and methylation patterns, to investigate tumor microenvironment (TME) biology. CtDNA consists of DNA fragments originating from tumors that are discharged into the bloodstream, encompassing tumor-specific modifications such as mutations, copy-number variations, and methylation patterns [146]. Because ctDNA shows the entire tumor burden, it can be used as a stand-in for residual disease and early treatment response.

In metastatic HER2-low breast cancer, longitudinal monitoring of ctDNA levels has demonstrated prognostic significance. A substantial investigation indicated that individuals whose ctDNA levels became undetectable following the initial cycles of therapy had markedly extended life. The same study also found significant differences in mutations, with TP53, PIK3CA, and ESR1 mutations being more common in HER2-low tumors than in HER2-0 cancers [57]. In general, ctDNA studies in BC show that patients with a greater baseline tumor burden, which is shown by a higher ctDNA fraction, and those who don’t clear their ctDNA early are more likely to have worse outcomes [146].

This indicates that tumors exhibiting elevated levels of inflammation or proliferation secrete greater quantities of DNA into the bloodstream. So, keeping an eye on ctDNA dynamics and looking at mutation patterns could give us indirect information about TME activity in HER2-low breast tumors. Exosomes, which are small membrane-bound vesicles secreted by both tumor and stromal cells, carry proteins, RNAs, and lipids that reflect the tumor microenvironment [23].

Liquid biopsy approaches, including circulating tumor DNA (ctDNA) and extracellular vesicles (exosomes), provide a non-invasive window into tumor-specific alterations such as mutations, copy number changes, and methylation patterns. Tumor-derived exosomes have been explored as biomarkers in breast cancer, with HER2-positive and CD24-positive exosome subsets from patient plasma revealing differentially enriched microRNAs that can distinguish early breast cancers from benign lesions with approximately 88% accuracy [147].

Exosomal microRNA profiles have potential as biomarkers for tumor presence and subtype, with HER2-low tumors possibly exhibiting a distinct EV miRNA signature. Beyond nucleic acids, tumor-derived exosomes carry immunomodulatory proteins. Liquid biopsy approaches, including ctDNA and exosomes, non-invasively capture tumor-specific alterations such as mutations, copy-number changes, and methylation patterns. Notably, exosomal PD-L1 can inhibit T-cell cytotoxicity, promoting tumor growth [21]. while other exosomal components, including cytokine receptors like IL-6R, can modulate immune cells and influence the tumor–immune balance [148]. Examining the molecular constituents of exosomes, such as PD-L1 protein, immunoregulatory microRNAs, or cytokine receptors, can serve as a biomarker indicative of the tumor microenvironment (TME) condition.

It has been suggested that circulating exosome levels of factors such as PD-L1 or HER2 serve as indicators of tumor burden and immune evasion [149]. In HER2-low breast cancer, researchers are examining exosomal markers indicative of the tumor microenvironment (TME), including EV-associated ERBB2 fragments and Treg-related miRNAs, as prospective biomarkers.

Liquid biopsy techniques provide a dynamic perspective of the tumor microenvironment (TME): circulating tumor DNA (ctDNA) reflects tumor cell turnover and the mutational landscape, while exosomes encompass immunological checkpoint proteins, cytokines, and microRNAs (miRNAs), which may be assessed as indications of TME activity [146].

## 7. Translational and Clinical Implications

### 7.1. Clinical Trials of HER2-Low BC Incorporating Microenvironmental Context

The success of Trastuzumab deruxtecan (T-DXd) in HER2-low metastatic BC (MBC) has transformed low-HER2 expression from a mere pathological descriptor into a therapeutically meaningful category. In the pivotal phase III DESTINY-Breast04 trial, 557 patients with previously treated HER2-low MBC (IHC 1+ or 2+/FISH−) were randomized 2:1 to receive T-DXd or physician’s choice chemotherapy. Treatment with T-DXd led to a median progression-free survival (PFS) of 9.9 months vs. 5.1 months with chemotherapy (hazard ratio [HR] 0.50; *p* < 0.001), and median overall survival (OS) of 23.4 vs. 16.8 months (HR 0.64; *p* = 0.001) [75]. Notably, the overall response rate (ORR) in the T-DXd group was 52.3%, considerably higher than historical rates with chemotherapy in similar populations [33]. The magnitude of benefit led to rapid regulatory adoption and reframing of HER2-low as a distinct therapeutic subtype [4].

However, despite this breakthrough, not all patients derive a durable benefit. Real-world and early translational analyses suggest that microenvironmental factors such as stromal density, extracellular matrix (ECM) architecture, vascular perfusion, and immune milieu heavily influence therapeutic efficacy and toxicity [33].

Given these observations, it is increasingly clear that future clinical trials should go beyond traditional endpoints (PFS, OS, ORR) and include translational sub-studies focusing on TME features: baseline and serial biopsies (spatial biology), liquid biopsies, perfusion/imaging biomarkers, and immune/stromal profiling [153].

Case Study 1 DESTINY-Breast04 real-world translational readouts: A 2025 translational follow-up reported that a subset of responders exhibited increased markers of immune activation and stromal remodeling post T-DXd, suggesting ADC-induced remodeling of the TME may contribute to sustained benefit [154]. Thus, while T-DXd validated HER2-low as druggable, the path forward must integrate the TME as a co-determinant of response, resistance, and long-term outcome.

### 7.2. Challenges in Trial Design: Heterogeneity, Dynamic TME, and Biomarker Limitations

Despite the clinical promise, translating TME–informed strategies into robust trials faces major obstacles, particularly the spatial and temporal heterogeneity of solid tumors, physical and biological delivery barriers, and limitations of conventional biomarkers.

#### 7.2.1. Spatial and Temporal Heterogeneity of the TME

Solid tumors (and specifically HER2-low breast cancers) are not homogeneous masses: they comprise micro-regions with diverse cellular compositions, stromal architecture, vascular patterns, and oxygenation states. Recent reviews characterize the TME as a dynamic “organ” in its own right, shaped by interactions among cancer cells, stromal cells, immune cells, and ECM [155].

Spatial transcriptomics, multiplex immunofluorescence, and single-cell analyses have revealed that different tumor zones may show opposite phenotypes, for example, an immune-“hot” niche with dense cytotoxic T-cells adjacent to a stromal/ECM-rich “cold” zone devoid of immune cells [156].

Consequently, a single baseline core biopsy may insufficiently represent the whole tumor, risking selection bias and misclassification. Overlooking this heterogeneity may lead to inaccurate biomarker-driven stratification in trials.

Moreover, TME evolves dynamically under therapeutic pressure. ADC-induced tumor cell death, payload release, and local cytotoxicity can alter stromal stiffness, vascular integrity, ECM composition, and immune infiltration changes that may occur between cycles, relapse, or after combination therapy [129].

Therefore, longitudinal sampling (on-treatment, post-treatment, at progression) coupled with spatial and molecular profiling is essential for capturing the evolving micro-environmental landscape, but poses logistic, ethical, and financial challenges.

#### 7.2.2. Physical and Biological Barriers to ADC Delivery and Distribution

Even when the target antigen (HER2) is present, several microenvironmental factors can restrict the effective delivery and distribution of ADCs, limiting their cytotoxic potential. Key barriers include:

Dense and cross-linked ECM/high stromal stiffness: The deposition of collagens, fibronectin, and other matricellular proteins by activated fibroblasts (CAFs) increases interstitial pressure, reduces interstitial fluid flow, and physically hinders diffusion of large molecules like ADCs [122].

Abnormal, poorly perfused vasculature: Tumor blood vessels are often tortuous, leaky, and inefficient, leading to heterogeneous perfusion, “cold” hypoxic zones, and inadequate delivery of ADCs, especially to tumor cores or mesenchymal-dense regions [77].

High interstitial fluid pressure and solid stress: Solid stress exerted by proliferating tumor mass plus stiff ECM compresses vessels, further impairing perfusion and drug convection [157].

Binding-site barrier effect and receptor heterogeneity: ADCs may bind heavily near well-perfused regions where HER2 density is adequate but fail to penetrate deeper or receptor-poor zones. In such areas, reliance on bystander effect (release of payload and diffusion to neighboring cells) may be inadequate if diffusion is limited or payload is rapidly cleared/metabolized [158].

A recent review argues that ECM stiffness is itself a therapeutic barrier, and reducing matrix rigidity can restore perfusion and drug diffusion, suggesting that matrix mechanics must be considered a core element of resistance in ADC therapy [157].

#### 7.2.3. Limitations of Conventional Biomarkers in HER2-Low Disease

Historically, HER2 status has been assessed by IHC and FISH, classifying tumors as HER2-positive, HER2-low, or HER2-0. However, this semi-quantitative, static classification fails to capture the complexity of ADC response, which depends on: antigen density, internalization kinetics, payload release, diffusion, microenvironmental barriers, and immune context. Indeed, the breadth of TME-mediated influences (stromal, vascular, immune) means that HER2 IHC alone is an insufficient predictive biomarker.

Recent reviews call for composite biomarker strategies, combining HER2 expression, ECM/vascular metrics, immune phenotyping, spatial transcriptomics, and dynamic markers (e.g., circulating tumor DNA, exosomes, perfusion imaging) [22].

Without such multi-dimensional biomarkers, clinical trials risk enrolling heterogeneous patients, some likely to benefit, others not, diluting efficacy signals, reducing statistical power, and misguiding treatment decisions.

### 7.3. Future Directions: Rational Combinations, Precision Immunotherapy and Microenvironment-Modulating Strategies

To overcome the described challenges and maximize benefit in HER2-low disease, future research must pursue rational combination regimens, next-generation ADC design, and integrative diagnostics treating the tumor and its microenvironment as co-targets.

#### 7.3.1. Rational Combination Regimens: ADCs + TME-Modulators (Vascular Normalization, ECM Remodeling, Immunotherapy)

Because the TME represents both a barrier and an opportunity, combining ADCs with agents that modulate the microenvironment could substantially improve efficacy and durability. Key strategies:

Vascular normalization: Use of low-dose or temporally scheduled anti-angiogenic agents (e.g., anti-VEGF/VEGFR) to “normalize” tumor vasculature, pruning immature/leaky vessels while improving structure and perfusion of remaining vessels has been shown to enhance delivery of cytotoxic agents [159]. Recent preclinical data indicate that combining vascular normalization with ADCs can significantly increase intratumoral drug concentration and improve tumor shrinkage compared to ADC monotherapy [160].

ECM remodeling/stromal modulation: Enzymatic or pharmacologic reduction in matrix stiffness (e.g., inhibition of cross-linking enzymes like lysyl oxidase LOX, or use of ECM-degrading enzymes) can reduce interstitial pressure and physical barriers, improving macromolecule penetration. A 2024 review summarized advances in targeting matrix stiffness to overcome drug resistance, highlighting this as a promising co-therapy [122].

Immune modulation/immunotherapy + ADCs: ADC-induced tumor cell death and antigen release can stimulate immunogenic cell death (ICD), potentially priming an anti-tumor immune response. When combined with immune checkpoint inhibitors (ICIs), especially in TME-permissive contexts (normalized vasculature, reduced ECM, improved perfusion), this may convert “cold” tumors into “hot,” yielding deeper and more durable responses [113].

Case Study 2: Preclinical proof-of-concept of ADC + vascular and ECM normalization: A 2023 study developed a bispecific ADC with enhanced penetration in desmoplastic tumors when preceded by vascular normalization and ECM-modulating agents, showing up to 2.5-fold increase in tumor drug concentration and a 60% higher tumor regression compared to ADC alone [161].

This combinatorial paradigm suggests that the optimal therapeutic regimen may not be a single “magic bullet,” but a multi-step, environment-aware sequence: first normalize vasculature or soften ECM, then deliver ADC, potentially followed by immunotherapy to exploit released antigens.

#### 7.3.2. Next-Generation ADC Design and Delivery Optimization

Parallel to combinatorial regimens, advancing the design of ADCs themselves is critical, particularly for complex, rigid microenvironments like those often seen in HER2-low breast cancer. Recent reviews highlight several innovations:

optimize linker–payload chemistry employing pH- or protease-sensitive linkers that release payload preferentially in the acidic, protease-rich TME; payloads designed for high membrane permeability to facilitate bystander effect.

Bispecific or multi-antigen targeting ADC targeting tumor cell antigens (e.g., HER2) but also stromal or ECM-associated antigens, to ensure delivery even in regions with heterogeneous HER2 expression [162].

Nanocarrier-based or ECM-penetrating platforms encapsulating ADCs or payloads in nanoparticles designed to navigate dense ECM, respond to TME cues (pH, enzymes), and release drug locally. Such platforms have shown enhanced penetration and efficacy in preclinical solid-tumor models [22].

For these advanced ADCs, early-phase clinical trials must integrate correlative endpoints: intratumoral drug concentration (immunofluorescence, mass spectrometry), spatial distribution, ECM/vascular changes, immune cell infiltration, and longitudinal outcome.

#### 7.3.3. Integrative Diagnostics: Spatial Biology, Perfusion Imaging, Liquid Biopsy and Computational Modeling

To implement precision medicine in HER2-low disease, companion diagnostics must evolve beyond IHC. A robust, integrative diagnostic pipeline should include:

Spatial biology (multiplex immunohistochemistry/immunofluorescence, spatial transcriptomics/proteomics, imaging mass cytometry) to map tumor cells, immune subsets, stromal components, ECM, vascular architecture, and hypoxia zones. Recent reviews argue strongly that spatial profiling is critical for understanding microenvironment-mediated resistance and for patient stratification [163].

Perfusion and structural imaging (DCE-MRI, contrast-enhanced MRI/CT, radiomics quantifying vascular morphology, vessel tortuosity, permeability, interstitial fluid pressure surrogate), novel radiomic biomarkers such as “Quantitative Tumor-Associated Vasculature” (QuanTAV) features have been shown to predict response and survival across multiple cancers, including breast cancer, when combined with clinical variables (AUC 0.63–0.71; HR for recurrence 1.25, 95% CI 1.08–1.44). These imaging-derived vascular phenotypes might help pre-select patients likely to benefit from ADCs or vascular-normalization preconditioning [164].

Liquid biopsy (ctDNA, exosomes, circulating immune or stromal markers) for non-invasive monitoring of clonal evolution, tumor burden, immune activation or suppression, emergence of resistance, and possibly ECM turnover or vascular remodeling markers (e.g., circulating collagen fragments, LOX activity). Reviews emphasize that integrating liquid biopsy with spatial and imaging data can provide dynamic, multidimensional insight into therapy response [22].

Computational modeling and AI-driven integration, given the complexity and multidimensionality of data (spatial, molecular, imaging, temporal), advanced computational frameworks (machine learning, graph neural networks, biophysical modeling) will be essential to integrate features, stratify patients into microenvironmental phenotypes (e.g., “vascular-normalized & immune-hot”, “ECM-dense & immune-cold”, “hypoxic & myeloid-rich”), and guide therapeutic decisions (monotherapy vs. combination, sequence, dose) [162].

Case Study 3—Radiomic vascular biomarkers as predictor of response: In a cohort of 371 BC patients treated with chemotherapy, pre-treatment QuanTAV radiomic scores (derived from contrast-enhanced MRI/CT) independently predicted recurrence-free survival (HR 1.25, 95% CI 1.08–1.44) and improved model AUC by 0.06–0.12 when added to clinical variables, supporting vascular morphology as a prognostic and predictive biomarker [165].

Implementing this integrative diagnostic approach in future HER2-low trials would allow precision stratification, adaptive therapy, and mechanism-informed endpoints, improving both efficacy and interpretability.

### 7.4. Advances in HER2-Low MBC: The Role of AI and ADCs

There have been big changes in the world of HER2-low metastatic breast cancer (MBC) in the last few years, especially since T-DXd was approved for HER2-low breast cancer. But more research, like important studies shown at ASCO 2025, is helping us learn more about how AI works in pathology and how ADC treatments work. Several studies at ASCO 2025 showed how helpful AI can be for finding HER2-low levels more accurately, especially in pathology images. AI models have done a better job of finding low HER2 expression levels than the old way of scoring. This could help doctors pick the best patients for ADC treatments [166]. This aligns with the growing trend toward integrating AI to address the challenges of subjective interpretation in HER2-low identification.

The DESTINY-Breast06 trial investigates the inaugural use of T-DXd in HER2-low, HR-positive, metastatic breast cancer and strongly supports early intervention. The study showed that this group of patients had a longer progression-free survival (PFS), which means that people who were thought to be ineligible for HER2-targeted therapies are now thought to be eligible [167].

The DAISY trial also looked at how the level of HER2 in breast cancer changes over time. It demonstrated that even minimal quantities of HER2 can indicate the efficacy of ADC treatments. This indicates the existence of a biological continuum rather than distinct subtypes If we want to help patients the most, we need to keep working on therapies that use biomarkers. This is what these important tests show.

## 8. Conclusions

HER2-low BC has emerged as a prevalent and therapeutically actionable subtype that challenges the historical binary classification of HER2 status. The clinical success of antibody–drug conjugates, particularly trastuzumab deruxtecan, has demonstrated that meaningful antitumor activity can be achieved even at low levels of HER2 expression, largely through payload-driven mechanisms and bystander killing. However, heterogeneity in response and the inevitability of resistance underscore that HER2 expression alone is insufficient to explain clinical outcomes. This review highlights the tumor microenvironment as a central determinant of ADC efficacy in HER2-low disease. Stromal architecture, extracellular matrix stiffness, vascular dysfunction, hypoxia, immune suppression, and metabolic reprogramming collectively shape drug delivery, payload distribution, immune engagement, and tumor cell survival. These factors operate in a spatially organized and dynamically evolving manner, creating permissive or restrictive niches that dictate therapeutic success or failure.

Importantly, emerging data indicates that these microenvironmental barriers are not static and can be transiently remodeled to enhance treatment response. Integrating TME-aware strategies into clinical development represents a critical next step. Rational combinations pairing ADCs with vascular normalization, stromal modulation, immunotherapy, or endocrine-informed sequencing offer a path to convert the TME from a barrier into a therapeutic amplifier. Achieving this goal will require a shift in trial design toward mechanism-informed endpoints, including spatial profiling, tumor pharmacokinetics, perfusion imaging, and longitudinal liquid biopsy monitoring, alongside conventional efficacy measures. From a clinical perspective, standardized and reproducible HER2 assessment, dynamic retesting across disease progression, balanced consideration of safety trade-offs beyond ILD, and multidisciplinary interpretation of biomarkers are essential to maximize benefit and minimize harm in real-world practice. Looking forward, the convergence of spatial biology, advanced imaging, liquid biopsy, and AI-driven data integration holds promise for precision stratification and adaptive treatment strategies. Drug delivery using nanoparticles has potential to bypass barriers in the tumor microenvironment that may limit efficacy of antibody drug conjugates (ADCs). Nanoparticles can help stabilize a drug, enhance diffusion through the dense extracellular matrix, and allow for controlled release of drug within the tumor in a controlled manner. While still primarily experimental in nature, combining nanotechnology with ADC therapy could improve drug distribution throughout a tumor and lead to enhanced responses from tumor cells; this may offer patients with HER2 low disease improved and enduring benefit from treatments they receive.

In conclusion, durable progress in HER2-low BC will depend on treating the tumor and its microenvironment as a unified, evolving ecosystem. By embedding microenvironmental biology into diagnostics, trial design, and therapeutic sequencing, it is possible to extend the transformative potential of ADCs and deliver more consistent, durable benefit to the large population of patients with HER2-low disease.

## Figures and Tables

**Table 2:** Synergistic strategies and tumor microenvironment (TME) interactions in enhancing ADC efficacy.

Component/Combination Strategy	Subcomponent/Marker	Mechanism of Synergy	Role of Tumor Microenvironment (TME)	Key Findings/Benefits	References
T-DXd + TME Proteases	Cathepsin L	Cleaves ADC linker extracellularly, enhancing payload diffusion and bystander killing.	Protease-rich TME increases extracellular linker cleavage.	Potentiates cytotoxicity in HER2-low and neighboring HER2-negative cells.	[97]
T-DXd + PD-1/PD-L1 Inhibitors	PD-1/PD-L1 Blockade	ADC-induced immunogenic cell death (ICD) promotes antigen release; checkpoint blockade sustains T-cell activation.	Converts “immune-cold” tumors to “hot” phenotype; Promotes CD8^+^ T-cell infiltration.	Improves CD8^+^ T-cell infiltration and durable tumor control.	[98]
T-DXd + CD47 Blockade	CD47 Blockade	Blocks “don’t-eat-me” signal; enhances macrophage phagocytosis of ADC-treated cells.	Reprograms suppressive myeloid environment.	Enhances macrophage phagocytosis of ADC-treated cells.	[99]
T-DXd + Endocrine Therapy	ER + HER2 Inhibition	Dual inhibition of the ER and HER2 pathways counteracts compensatory signaling in HR+ HER2-low tumors.	CAFs modulate ER signaling and resistance, influencing tumor survival and therapy efficacy.	Dual inhibition of the ER and HER2 pathways enhances efficacy in HR+ HER2-low tumors.	[4]
ADC + VEGF Inhibitor	VEGF Blockade	Vascular normalization enhances ADC delivery and reduces interstitial pressure.	Improves tumor perfusion, reduces hypoxia, and increases drug penetration.	Improves ADC delivery by enhancing vascular normalization.	[100]
ADC + CAF-Targeting Agents	Collagen + Hyaluronan Degradation.	Degradation of ECM components reduces physical barriers to drug delivery.	Decreases stromal density and improves ADC diffusion into the tumor.	Enhances ADC delivery by reducing ECM barriers.	[27]
Adaptive Immune Cells	CD8^+^ T-cells (GZMB, PRF1, IFNG)	Cytotoxic clearance post-ADC.	Patchy islands near cold stroma; role in targeting tumor cells.	Increased tumor clearance, potentially aiding ADC efficacy.	[101]
Adaptive Immune Cells	CD4^+^ Th1 vs. Th2	Th1 supports CD8; Th2 promotes inflammation.	Intermixed with CD8; Th2 in fibrosis and immune regulation.	Th1 cells enhance CD8 T-cell activity; Th2 cells encourage inflammation.	[102]
Innate Immune Cells	NK Cells (CD56dim, CD16)	ADCC of opsonized cells.	Reduced NK activity in desmoplasia/hypoxia.	Reduced NK cell activity limits immune response in TME.	[103]
Myeloid Compartment	SPP1^+^ M2 TAMs	ECM remodeling, immunosuppression.	Collagen-rich stroma supports M2 phenotype and promotes immune suppression.	Immunosuppressive role in TME, hindering anti-tumor immune response.	[80]
Myeloid Compartment	MDSCs (PMN/M)	T cell suppression, fibrosis.	Stromal tracks hinder T-cell infiltration and contribute to fibrosis.	MDSCs promote fibrosis and immune suppression in the TME.	[104]
Regulatory Compartment	Tregs (FOXP3^+^)	blunt effectors.	Immune-excluded niches restrict T-cell activity and tumor clearance.	Suppression of effector T-cell function in immune-excluded regions.	[104]

**Table 3:** Key biomarkers from the TME in HER2-low breast cancer.

Biomarker Category	Mechanistic Role	Prognostic/Predictive Value	Detection Methods	References
TIL Density	Represents immune infiltration; higher TILs indicate stronger antitumor response, with CD8^+^ T cells driving cytotoxicity.	High TILs (>10%) correlate with improved disease-free survival (DFS) in HER2-low tumors; lower TILs indicate weaker immune response and poorer outcomes.	Multiplex immunohistochemistry (IHC), spatial transcriptomics, single-cell RNA sequencing (scRNA-seq).	[6,52,53,143]
Immune Gene Signatures (e.g., PD-L1, TIGIT)	PD-L1 suppresses T-cell activity; TIGIT inhibits immune activation; interferon/T-cell signatures reflect immune-hot vs. cold TME.	Lower PD-L1/TIGIT in HER2-low/ER+ tumors suggest reduced immunogenicity; high signatures predict better response to immunotherapy/ADCs.	Gene expression profiling (e.g., RNA-seq), spatial transcriptomics, bulk transcriptomics.	[52,53,128,144]
Spatial Features (e.g., TLS Density, CAF Subtypes)	TLS are immune aggregates promoting antitumor responses; CAF subtypes (e.g., iCAF, myCAF) remodel ECM and suppress immunity.	High TLS density links to better immune activation and survival; CAF-rich zones indicate immune exclusion and resistance.	Spatial transcriptomics (e.g., Visium), multiplex immunofluorescence, imaging mass cytometry.	[111,125,145]
ctDNA Mutations (e.g., TP53, PIK3CA, ESR1)	Reflects tumor burden and mutational landscape; mutations drive resistance and indicate aggressive biology.	High baseline ctDNA or lack of clearance predicts poorer survival; common in HER2-low vs. HER2-zero.	Liquid biopsy (next-generation sequencing), digital PCR.	[57,146]
Exosomal Markers (e.g., PD-L1, miRNAs, Cytokine Receptors)	PD-L1 on exosomes inhibits T-cells; miRNAs/cytokine receptors (e.g., IL-6R) modulate immune suppression and signaling.	High exosomal PD-L1 correlates with immune evasion and tumor burden; specific miRNAs distinguish early BC subtypes.	Liquid biopsy isolation (ultracentrifugation, immunoprecipitation), RNA/protein profiling (qPCR, ELISA).	[21,147,148,149]

## Data Availability

No new data were created or analyzed in this study. Data sharing is not applicable.

## Abbreviations

ADC: antibody-drug conjugate; ADCC: antibody-dependent cellular cytotoxicity; ADCP: antibody-dependent cellular phagocytosis; AI: artificial intelligence; AKT: protein kinase B; ASCO: American Society of Clinical Oncology; BBB: blood-brain barrier; BC: breast cancer; CAF: cancer-associated fibroblast; CAP: College of American Pathologists; CCL: C-C motif chemokine ligand; CD: cluster of differentiation; CDK: cyclin-dependent kinase; CNS: central nervous system; CT: computed tomography; CTCAE: Common Terminology Criteria for Adverse Events; ctDNA: circulating tumor DNA; CXCL: C-X-C motif chemokine ligand; DAMP: damage-associated molecular pattern; DCE-MRI: dynamic contrast-enhanced magnetic resonance imaging; DCIS: ductal carcinoma in situ; DFS: disease-free survival; ECM: extracellular matrix; EMT: epithelial-mesenchymal transition; ER: estrogen receptor; ERBB2: Erb-B2 receptor tyrosine kinase 2; EV: extracellular vesicle; FAK: focal adhesion kinase; FAP: fibroblast activation protein; FISH: fluorescence in situ hybridization; FOXP3: forkhead box P3; G-CSF: granulocyte colony-stimulating factor; GPR81: G protein-coupled receptor 81; GZMB: granzyme B; HER2: human epidermal growth factor receptor 2; HIF: hypoxia-inducible factor; HR: hormone receptor; ICD: immunogenic cell death; IFNG: interferon gamma; IFN: interferon; IFP: interstitial fluid pressure; IHC: immunohistochemistry; IL: interleukin; iNOS: inducible nitric oxide synthase; IO: immuno-oncology; ISH: in situ hybridization; IV: intravenous; LAG-3: lymphocyte-activation gene 3; LDHA: lactate dehydrogenase A; LOX: lysyl oxidase; LRRC15: leucine-rich repeat containing 15; M2: M2-polarized macrophage; MAPK: mitogen-activated protein kinase; MBC: metastatic breast cancer; MCT: monocarboxylate transporter; MDSC: myeloid-derived suppressor cell; MMP: matrix metalloproteinase; MRI: magnetic resonance imaging; MRS: magnetic resonance spectroscopy; mTOR: mammalian target of rapamycin; myCAF: myofibroblastic cancer-associated fibroblast; NK: natural killer; ORR: overall response rate; OS: overall survival; PCR: pathologic complete response; PD-1: programmed cell death protein 1; PD-L1: programmed death-ligand 1; PFS: progression-free survival; PI3K: phosphoinositide 3-kinase; PIK3CA: phosphatidylinositol-4,5-bisphosphate 3-kinase catalytic subunit alpha; PK: pharmacokinetics; PMN: polymorphonuclear; PRF1: perforin 1; PTEN: phosphatase and tensin homolog; QOL: quality of life; RAS: Ras family; RC48: disitamab vedotin; RFS: recurrence-free survival; RWD: real-world data; SERD: selective estrogen receptor degrader; SG: sacituzumab govitecan; SHG: second harmonic generation; SPP1: secreted phosphoprotein 1; SRC: Src family kinase; STAT3: signal transducer and activator of transcription 3; STING: stimulator of interferon genes; TAZ: transcriptional coactivator with PDZ-binding motif; TAM: tumor-associated macrophage; TAN: tumor-associated neutrophil; T-DM1: trastuzumab emtansine; T-DXd: trastuzumab deruxtecan; TGF-β: transforming growth factor beta; TIGIT: T cell immunoreceptor with Ig and ITIM domains; TIL: tumor-infiltrating lymphocyte; TIM-3: T cell immunoglobulin and mucin domain-containing protein 3; TLS: tertiary lymphoid structure; TLR4: toll-like receptor 4; TME: tumor microenvironment; TNBC: triple-negative breast cancer; TREG: regulatory T cell; TROP2: trophoblast cell-surface antigen 2; VEGF: vascular endothelial growth factor; VEGFR: vascular endothelial growth factor receptor; YAP: Yes-associated protein.
